# “I Have Everything to Win and Nothing to Lose”: Patient Experiences of Mobilization Out of Bed Immediately After Abdominal Surgery

**DOI:** 10.1093/ptj/pzaa168

**Published:** 2020-09-17

**Authors:** Anna Svensson-Raskh, Anna Schandl, Ulrika Holdar, Monika Fagevik Olsén, Malin Nygren-Bonnier

**Affiliations:** Department of Neurobiology, Care Sciences and Society, Division of Physiotherapy, Karolinska Institutet, Alfred Nobels Allé 23, 141 83, Stockholm, Sweden; and Department of Allied Health Professionals, Functional Area Occupational Therapy and Physiotherapy, Karolinska University Hospital, Stockholm, Sweden; Department of Molecular Medicine and Surgery, Karolinska Institute and Department of Anesthesiology and Intensive Care, Södersjukhuset, Stockholm, Sweden; Department of Allied Health Professionals, Functional Area Occupational Therapy and Physiotherapy, Karolinska University Hospital; Department of Neuroscience and Physiology, Division of Health and Rehabilitation/Physical Therapy, Sahlgrenska Academy, Gothenburg University, Göteborg, Sweden; and Department of Physiotherapy, Sahlgrenska University Hospital, Göteborg, Sweden; Department of Neurobiology, Care Sciences and Society, Division of Physiotherapy, Karolinska Institutet and Department of Allied Health Professionals, Functional Area Occupational Therapy and Physiotherapy, Karolinska University Hospital

## Abstract

**Objective:**

Early mobilization is advocated for patients going through abdominal surgery; however, little is known about the patient experience of being mobilized immediately after surgery. The purpose of this study was to explore patient experiences of mobilization immediately after elective abdominal cancer surgery.

**Methods:**

This interview study used qualitative content analysis. With the use of purposeful sampling, a total of 23 participants who had been mobilized immediately after abdominal surgery were recruited at a university hospital in Stockholm, Sweden. Individual face-to-face interviews were conducted within 1 to 4 days after surgery and took place at the surgical ward where the participants were treated. A semi-structured guide was used. All interviews were audio recorded and transcribed verbatim.

**Results:**

The content analysis revealed 3 categories that emerged into 1 overarching theme: “to do whatever it takes to get home earlier.” The participants experienced that mobilization out of bed had an impact on their physical and mental well-being. Motivation and the experiences of themselves and others were factors that affected patient attitudes toward early mobilization. Preparation and competent caregivers were emphasized as important factors that enabled the patient to feel safe and confident during mobilization.

**Conclusions:**

Patients experienced mobilization as an important part of the care that had an impact on recovery and well-being, physically as well as mentally, both immediately and over time.

**Impact:**

As this is the first study to our knowledge to investigate patient experiences of mobilization immediately after abdominal surgery, this information can be used to support the development of early mobilization protocols in hospital settings.

##  

Early mobilization is advocated for patients going through elective abdominal surgery. Mobilization as an intervention is suggested to be a key component to improving respiratory function and preventing the risk for postoperative complications, such as pneumonia, respiratory insufficiency, and thromboses.^1^^,^^2^ In Sweden, patients are recommended to mobilize as early as possible, preferably the day of surgery, that is postoperative day 0 (POD 0), and if possible, out of bed. However, in clinical practice, most of the patients are out of bed the day after surgery (POD 1). This is possibly due to the lack of a common definition of “early mobilization.” According to guidelines for Enhanced Rehabilitation After Surgery (ERAS), mobilization is recommended out of bed at the day of surgery (POD 0).^2^^,^^3^ ERAS is a multimodal and multidisciplinary concept consisting of a combination of pre-, per-, and post-surgical interventions, such as mobilization, energy intake, and individual anesthetic and analgesic arrangements with the overarching aim to facilitate/enhance patient recovery after surgery.^3^ In a recent systematic review, it was concluded that the effect of early mobilization protocols could not be determined, mostly because the included studies evaluated the outcome at different time points.^4^ In addition to the lack of evidence, most studies were of poor methodologic quality and had conflicting results.^4^ Moreover, the adherence to different mobilization protocols as well as to the ERAS recommendations regarding mobilization has not been reported and is therefore still unknown.^4–6^

Even though mobilization out of bed is highly recommended, it remains unclear how patients experience mobilization. Previous qualitative research has mainly focused on patient experiences of protocolled package interventions, such as in the ERAS concept, by interviewing patients about the care process and not about mobilization as an intervention.^7–12^ Most patients have been found to appreciate interventions that lead to a short hospital stay.^8^^,^^9^^,^^12^ Moreover, studies have shown that patients want to take control of their situation and their body as a way of restoring their autonomy after surgery.^7^^,^^8^^,^^10^^,^^11^^,^^13^ To be able to do that, they need information about the entire process they are going through.^7^^,^^11^^,^^13^^,^^14^ Studies have revealed that the underlying cause of surgery, the surgery itself, the postoperative period, and fear of pain are associated with anxiety during the first period after surgery.^7^^,^^10^^,^^12^

It is important that caregivers and patients collaborate closely to achieve the best possible outcome.^9^^,^^11^^,^^13^ Aasa et al^13^ described patients’ experiences with preoperative information prior to colorectal surgery and suggested that information created a sense of trust and preparedness, making them aware of their responsibilities and involved in their own care. However, Aasa et al^13^ highlighted the need for caregivers and patients to be aware of each other’s responsibilities. Norlyk et al^11^ explored patients’ experiences of fast-track programs after colonic surgery, and described the participants who strived to regain their health as being cooperative and compliant with the expectations of the professionals. Caregivers need to gain a deeper understanding of what the intervention means to patients and identify factors important prior to, during, and after mobilization in order to prepare, help, and assist patients in the best way. This makes it easier for the patient and the caregiver to interact in the recovery process after surgery. Therefore, we conducted a study about exploring and describing patient experiences of mobilization by including and interviewing patients who had participated in a randomized controlled trial (RCT) evaluating the respiratory effect of mobilization out of bed within 2 hours after elective abdominal surgery. (Svensson-Raskh A, Nygren-Bonnier M, Schandl A, Ståhle A, Fagevik Olsén M, unpublished data, 2020.) The term immediate mobilization was used to reflect mobilization within 2 hours after surgery.

## Methods

To address the aim of the present study, the authors used a qualitative approach where content analysis, as described by Graneheim and Lundman,^15^ was used as a method. To maximize transparency and to increase trustworthiness of the results, the authors described a step-by-step process.^15–19^

### Participants and Sampling

Participants were recruited from an RCT investigating the respiratory effect of mobilization after elective abdominal surgery. In brief, the RCT enrolled adult patients at a postoperative recovery unit who underwent elective abdominal surgery (with an anesthetic duration exceeding 2 hours) because of cancer at a university hospital in Stockholm, Sweden, between January and September 2017. The participants were randomly assigned to either mobilization out of bed to sitting in a chair (within 2 hours after surgery), to mobilization (as described) + breathing exercises, or to the control group (Svensson-Raskh A, Nygren-Bonnier M, Schandl A, Ståhle A, Fagevik Olsén M, unpublished data, 2020)

The criteria for inclusion in the present qualitative study were attendance in the RCT and randomization to 1 of 2 intervention groups: mobilization or mobilization + breathing exercises. Participants also had to understand and speak Swedish. No exclusion criteria were applied. All participants gave written informed consent. The inclusion started March 1 and ended in June 30, a period of 4 months. The participants were purposively sampled during the inclusion process to ensure a maximum variation of age, sex, American Society of Anesthesiologists physical status classification,^20^ surgery, anesthetic time, and total time of mobilization to achieve as broad a range of experiences as possible.^16^^,^^17^ There was a dialogue between the interviewer and the researchers about the quality of the interviews during the ongoing recruitment to ensure enough richness to respond to each research question.^21^ After interviewing a total of 20 patients, nothing notably new related to the aim emerged, 3 more patients were included, and then inclusion stopped.^21^ Thus, a total of 23 patients were recruited and interviewed: 13 women and 10 men. The ages ranged from 38 to 80 years (median age 65 years). The demographics are presented in Table 1.

**Table 1:** Characteristics of the Participants With Experience of Immediate Mobilization at the Postoperative Recovery Unit (N = 23)*^a^*

**Id No.**	**Sex**	**Age (Range)**	**ASA PS Classification** ^***^b^***^	**Type of Surgery Performed**	**Length of Anesthesia, h (Range)**	**Total Time Sitting Up, h.min**
1	Male	70–80	3	RALC	6–7	2.20
2	Female	60–70	2	RALH	4–5	1.50
3	Female	50–60	2	Open abdominal*^c^*	2–3	1.20
4	Female	70–80	3	Open abdominal gynecological*^d^*	3–4	2.25
5	Female	50–60	3	Open abdominal gynecological*^d^*	3–4	2.15
6	Female	40–50	1	Open abdominal gynecological*^d^*	5–6	1.10
7	Female	60–70	2	RALH	2–3	1.35
8	Female	70–80	3	RALH	2–3	3.25
9	Male	60–70	3	Open abdominal*^c^*	2–3	0.35
10	Male	80–90	3	RALC	6–7	3.00
11	Male	60–70	3	RALC	5–6	2.45
12	Male	80–90	3	RALC	5–6	0.30
13	Male	50–60	3	Open abdominal*^*^c^*^*	2–3	2.50
14	Male	70–80	3	RALC	7–8	1.25
15	Female	60–70	1	Open abdominal gynecological*^d^*	5–6	1.10
16	Male	60–70	2	Open abdominal^*c*^	4–5	1.20
17	Female	50–60	1	Open abdominal gynecological*^d^*	4–5	1.20
18	Male	30–40	2	Open abdominal*^*^c^*^*	2–3	0.20
19	Female	50–60	3	Open abdominal gynecological*^d^*	2–3	4.10
20	Male	70–80	2	RALC	8–9	3.15
21	Female	70–80	2	Open abdominal gynecological*^d^*	2–3	2.20
22	Female	50–60	2	Open abdominal gynecological*^d^*	3–4	2.10
23	Female	80–90	2	Open abdominal gynecological*^d^*	3–4	0.55

*^a^* GIST = gastrointestinal stromal tumor; RALC = robot assisted laparoscopic cystectomy (including Bricker deviation and removal of lymphatic nodes in pelvic region); RALH = robot assisted laparoscopic hysterectomy (including salpingo-oophorectomy and removal of lymphatic nodes in pelvic and para-ortal region).

*^b^* American Society of Anesthesiologists physical status classification system, where 1 = a normal healthy patient, 2 = a patient with mild systematic disease, and 3 = a patient with severe systematic disease, 4 = a patient with severe systematic disease that is a constant threat to life, 5 = a moribund patient who is not expected to survive without the operation, and 6 = a declared brain-dead patient whose organs are being removed for donor purposes.

*^c^* Sarcoma or GIST (in some cases involving hemicolectomy, ventricular resection).

*^d^* Open abdominal gynecological surgery (involving hysterectomy, salpingo-oophorectomy and removal of lymphatic nodes in pelvic and para-ortal region).

**Table 2:** Examples of the Analysis Process

**Examples of Meaning Units**	**Condensation**	**Codes**	**Subcategory**	**Category**
It sort of felt easier in my chest. That’s what I mostly felt. Then I got tired, but I could feel that it was sort of, that it was better for my lungs and stuff.	It felt easier in my chest and was good for my lungs, then I got tired.	Effects	The physical and mental aspects	The impact of mobilization
I wasn’t in pain, but I vomited when I sat up. Then it was a little confusing with breathing. It felt better to breathe sitting up in the chair.	Despite vomiting, sitting in the chair was good for breathing.	Symptoms and effects		
It was positive to sit! Then I could see what happened with my pulse, blood pressure, I had an overview of my surroundings and could see what time it was. I did not have to just lie in bed staring into the ceiling.	It was positive to sit, get an overview of the surroundings and their own situation.	Effect		
Of course, I was in pain, but I was a little surprised that I did not have more pain.	Felt pain but surprised that it did not hurt more.	Experience		
When I got to the bed, I felt very happy when I could just lie down.	Very happy to lie down.	Reward	To achieve a goal	
It’s not good to lie there, your bowels shrivel somehow, I wanted them to move, so I could be normal.	Not good to just lie there, wanted their bowels to move.	Motivation	Attitudes and motivational factors	Experiences and motivational factors
Yeah, I could have my feet on the floor, and they probably helped me to move and pulled the chair forward and stuff. Yeah, it felt good.	They helped me, pulled the chair forward.	Help (assistance)	The importance of professional and competent caregivers	To feel safe and be confident with the mobilization process
I felt safe and secure. It was nice to have someone there taking care of me, that probably made me feel safe with what I was supposed to do, I think.	Safe and secure, someone took care of me.	Safe		

### Data Collection

Face-to-face interviews were conducted by 2 physiotherapists, U.H. and A.S.R., both with clinical experience of mobilizing patients postoperatively and experience in the qualitative field of research.

To ensure participant experiences of early mobilization were fresh in mind, all interviews were carried out within 1 to 4 days after surgery. However, 1 participant was discharged from the hospital before the planned interview and was therefore interviewed 2 weeks after surgery.

**Figure 1:**
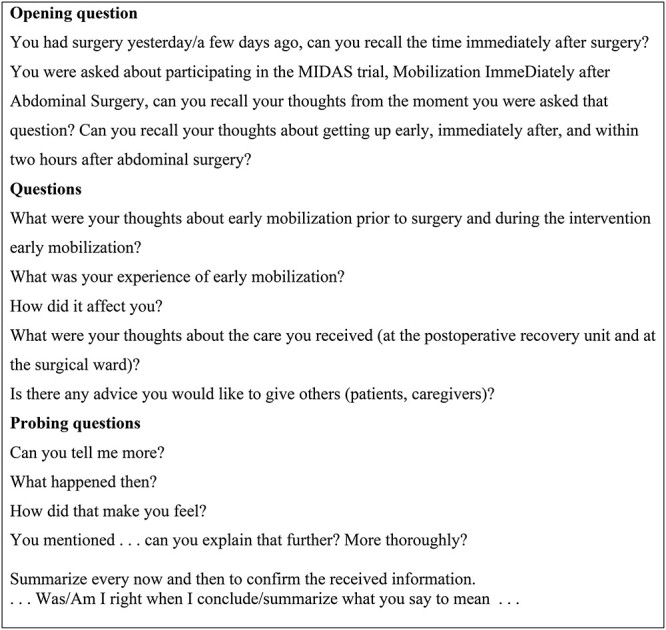
Interview guide: short form.

The individual interviews took place at the surgical ward, mostly in the participants’ private rooms. The interviews followed a semi-structured guide with set topics, such as thoughts about mobilization, inconveniences, experiences of mobilization, assistance and support from caregivers, and the future (Fig. 1). The guide was tested during a pilot interview; however, the only aspect of the guide that was changed was that questions were formulated as more open-ended and probing to facilitate a broader description of participant experiences.^16^^,^^22^ Interviews lasted 11 to 54 minutes, with an average interview time of 22 minutes, and were audio-recorded then transcribed verbatim by a person experienced in the method but with no further interest in the study. Each interview was assigned a random number, and all personal data were removed. Thereby, all participants were anonymized in the transcript and in the analysis.

### Data Analysis

The analysis process followed the content analysis described by Graneheim and Lundman.^15^ The transcribed interviews were then read and reread by the first author (A.S.R.) to achieve a sense of the whole. Each text was read with the purpose of the study in mind whereby meaning-units were identified and marked.^15^^,^^18^ The meaning-units were then condensed, abstracted, and labeled with codes, thus maintaining the core of the text. Then M.N.B. contributed to the process by discussing and validating the material and its connection to the meaning units and the codes.^15^^,^^19^ The next step was to organize the codes into categories and subcategories by comparing and abstracting the codes so that they could be sorted intocategories and subcategories based on similarities.^15^^,^^18^ Thereafter, the process and categorizations were discussed and validated by all authors, and the sub-categories were merged from 12 to 7 linked to 3 main categories.^23^^,^^24^ The subcategories and categories were accompanied by illuminating quotations from the participants to enrich the material. Finally, while viewing the codes, an underlying meaning emerged into an overarching theme of subcategories and categories, thereby corroborating the analysis to the general research topic.^15^  Table 2 displays the transparency of the analysis process.

The researchers’ preunderstandings in the field of qualitative research and methods are broad (A.S., M.F.O., and M.N.B.) as well as their clinical experience in mobilization of critically ill patients at intensive care units (A.S., A.S.R., and M.N.B.) and at postoperative recovery units and surgical wards (A.S.R. and M.F.O.). The research group consisted of 3 physiotherapists (A.S.R., M.F.O., and M.N.B.) and 1 nurse, all used to and experienced in working as a team with mobilization of patients immediately after surgery, which enriched their interpretation during the process of data analysis.^23^^,^^24^

### Ethical Considerations

All participants provided written informed consent prior to study inclusion. The study received ethical approval from the regional board of ethics in Stockholm (Dnr: 2015/703/31–31) and followed the 1964 Declaration of Helsinki and its later amendments regarding the involvement of human participants. Participation confidentiality was obtained during the analysis process using identity coded transcripts.

### Role of the Funding Source

The funders played no role in the design, conduct, or reporting of this study.

## Results

The theme “to do whatever it takes to get home earlier” emerged from participant experiences of mobilization out of bed immediately after elective abdominal surgery because of cancer. The participants described factors such as preparedness along with professional and competent caregivers that created a feeling of safety and confidence prior to and during early mobilization. Experiences and motivational factors, their own and other persons, emerged as important to facilitate early mobilization. The impact of mobilization, physically and mentally, was described as important since the patients experienced that mobilization made it easier to breathe, and helped them to wake up and to regain their body and mind again after surgery. They described getting out of bed as a goal because they wanted to be in charge of their own situation and wished for early discharge from the postoperative recovery unit and surgical ward to reach their main goal, which was returning home.

The overarching theme is illustrated by the following patient quotation:

I was hoping that it would help me even at this stage. That it would speed up my mobilization in general . . . and I felt like that I had everything to win and nothing to lose. —P21

**Figure 2:**
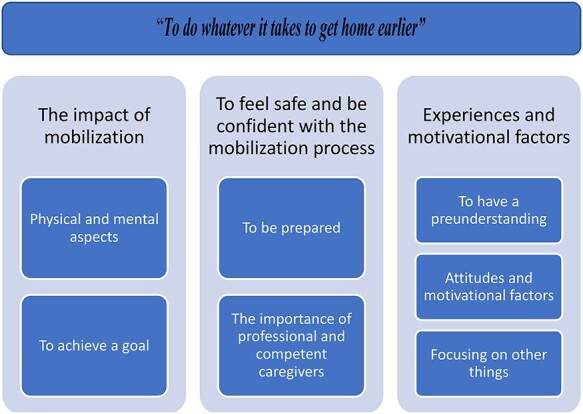
The overarching theme and the 3 categories followed by each subcategory that emerged in the content analysis of patient experiences of immediate mobilization after abdominal surgery.

The analysis resulted in 3 categories and 7 subcategories, each of which is presented in the text below and displayed in Figure 2.

### The Impact of Mobilization

This category emerged from the subcategories concerning the direct effects of mobilization out of bed immediately after surgery along with the experience of achieving a goal by being mobilized. The participants’ physical and mental experiences of mobilization are interconnected in this category, and the impact of the effect of mobilization could appear immediately, or later, after sitting up for a while. However, patients also experienced mobilization as difficult to recall at the postoperative recovery unit.

The following category comprises 2 sub-categories.

#### Physical and Mental Aspects

This sub-category comprises the overall physical and mental effects of mobilization out of bed immediately after abdominal surgery. Prior to mobilization, participants experienced dizziness and tiredness and lacked a perception of time. The participants said that rising to a sitting position cleared their mind and made them more alert and oriented to the day, the time, and the person next to them as they got an overview of their surroundings and could see the clock on the wall. In other words, the patients became more aware of what was going on with themselves and how their body and mind actually felt. They experienced communicating with caregivers, reading a paper, and drinking as easier when sitting up. They also experienced breathing as easier while sitting up compared with lying in bed. Pain and tiredness prior to mobilization were described as worse than expected and also less than expected. Mobilization did not increase pain; on the contrary, physical motion was experienced as reducing and gaining control of pain. Nausea could be worsened by mobilization but also the opposite. Nevertheless, the patient experience was that it was better to vomit in a sitting position out of bed rather than in bed. Mobilization and sitting up were experienced as the cause of physical tiredness, a recognizable tiredness compared with anesthetic tiredness. While mobilizing and sitting up, patient focus was on something other than pain, nausea, and surgery. Patients described the sitting position as regaining contact with their body.

…so, I thought, I got so darned tired when I stood up. And then I thought like this, no, I’m going to say that I don’t want to do this, but then that feeling passed a little bit, so it was entirely okay and then I felt that when I got to sit in a chair, it was much easier to breathe and that kind of stuff. And it wasn’t that hard either when I sat well, you know. So, I thought it went well, better than what I had actually thought. That’s what I think. —P6

Being mobilized at the postoperative recovery unit was sometimes difficult to remember. However, patients explained that this did not bother them; they just assumed that everything went the way it was supposed to.

No, I’m too dizzy to remember that. No, I really do not remember. —P10

#### To Achieve a Goal

To mobilize and be able to get out of bed early was experienced as satisfactory and described as a goal to reach, or a “box to tick” after completed mobilization. Irrespective if the participant sat up for a longer or a shorter period of time, bedrest after mobilization was experienced as a type of reward, where the patient could just let their body rest and relax again.

…and then you got to go back to bed, and it was so nice . . .Yeah, it was like oh my how nice, now I’m here again, I don’t need to think, just let my body lie where it lies. —P8

I hope that I can perform, so that I am being good . . . I want to get a gold star. —P23

### To Feel Safe and Be Confident With the Mobilization Process

This category relates to the participants’ feeling of safety and trust in themselves and the caregivers prior to, during, and after the intervention. They felt that they were prepared for the mobilization procedure. They said that the team, the nurse, and the physiotherapist worked closely together and with the patient; were experienced and calm; and had the competence and the routines for mobilizing patients at the postoperative recovery unit.

The category below comprises 2 sub-categories.

#### To Be Prepared

The participants underlined the importance of information about the mobilization process, how it was to be done, and what was expected of them. Information about how their body could react during mobilization, both negative responses, such as dizziness or nausea, as well as positive responses, such as easier to breath or a feeling of alertness, was also something they addressed as important to know in advance. They also appreciated simple recommendations about how to counteract low blood pressure or dizziness, such as pedaling with their feet prior to and during mobilization. All of this was described as creating a feeling of trust and safety prior to and during the intervention; hence, they knew and were prepared for each step, from sitting on the edge of the bed to transferring to the chair and back to the bed again.

Yeah, like she told me what would happen, that we would do this, and we would do that. And then she just did what she had said before. And I thought it was so darned good . . . But that it felt very safe and good, I got to admit that. I think that is important. —P26

…she put those support hose on me …so that I wouldn’t slip, naturally….and then sit on the edge of the bed, carefully…That’s how you are to step the whole time and so over on to a chair. —P11

#### The Importance of Professional and Competent Caregivers

Prior to, during, and after mobilization, the participants described a feeling of safety and trust in the caregivers. The participants sensed that the caregivers were present, professional, and with sufficient knowledge of and competence in mobilizing patients at the postoperative recovery unit. They experienced that the caregivers responded promptly when they felt dizzy, had nausea or pain, and were alert with medications, which helped them to remain calm. Participants then said that they felt safe and able to mobilize out of bed in a situation they never thought would have been possible.

Yeah, first I thought dear me, do they really know what they’re doing? But then as I said A had a very confident way and she also said that, you’re in good hands, I can look at you and see whether you can do it or not. That’s about how she explained it. So, if I can or if I can’t, we’ll just stop. That’s about how she explained it, so I had complete faith in her. —P21

I thought you were really attentive and so confident and knew what you were doing, you know, so I thought it worked well, everything. —P2

It also emerged that the participants felt safe if the caregiver was familiar to them.

There I felt, possibly in general, that to have a person you recognize feels good compared with if new people come all the time. —P13

### Experiences and Motivational Factors

Participant preunderstandings and motivational factors, negative as well as positive, about mobilization emerged in this category. However, it also emerged that focus was sometimes not on the intervention itself.

The following category comprises 3 sub-categories.

#### To Have a Preunderstanding

A preunderstanding of the physical and mental consequences of mobilization was expressed as participants’ prior experiences and was based on caregivers saying that mobilization out of bed was important. Friends and family also stressed the importance of early mobilization after surgery. The preunderstanding was that mobilization reduces the risk for postoperative complications such as thrombosis and pneumonia.

…but they have a lot of that these days that you are, you know, to get up right away and . . . No, it’s never good to lie in bed . . . and the earlier you hop up out of bed, the better it will be. Get started with your belly and breathing and blood pressure and everything. The whole works. —P15

But everyone says that you are to move as much as possible, everyone says that, you know. So, you’re sort of pushed. Because it’s important. So, you shouldn’t think that anything is going to open up or break or . . . —P7

#### Attitudes and Motivational Factors

Motivational factors for some participants were experienced as non-motivational for others. Participants said that they were motivated to mobilize and move their body as a way of reducing pain, dizziness, nausea, and risk for postoperative complications. In contrast, participants were unmotivated to move their body because of the fear of getting surgical complications, putting a strain on their wound, pain, nausea, or fear of falling. They said that they had been through comprehensive surgery and were maybe too fragile to consider mobilization. However, the participants’ attitude towards physical activity overall emerged as a motivational factor, since they wanted to stay active, even though they were recovering from surgery.

I’m one of those who does things and wants them done. And then they’re done and over. So, isn’t there anything that we can do later or the next day? That doesn’t work. I just go ahead and do it, we just went ahead and did it. —P1

Yeah, but as soon as you feel that you want to move, should move around, that’s when you should do it, I believe. Even if it hurts. Otherwise, it’s easy to remain lying in bed and thinking, yeah, they’ll take care of me, so this will be good, but you have to be active yourself. . . It’s better that I move around. That’s how I try to think. —P4

#### Focusing on Other Things

Participants said that they did not have any expectations concerning mobilization since their only focus was to manage the surgery and cope with their cancer diagnose. The postoperative period was described as a period of waiting for the surgeon to inform them about how the surgery had gone, to find out if the cancer had been completely removed. Since they had no expectations about mobilization, participants said that they put themselves in the hands of the caregivers and trusted their preunderstandings in the matter and trusted staff to handle nausea, pain, and dizziness.

You don’t think about anything, you’re not capable of thinking when you’re full of morphine and whatever it’s called . . . I just wondered how many organs I still had in my belly. —P3

### Additional Results

Present section describes findings not directly related to the aim of the study.

The participants experienced a gap between the postoperative recovery unit and the surgical ward in terms of mobilization and pain. Once mobilized at the recovery unit, they expressed a willingness to get out of bed when they had arrived at the surgical ward. However, at the surgical ward, they perceived that pain control was not as efficient as at the postoperative unit. Lack of staff in the vicinity contributed to a delay in mobilization since patients had to wait for assistance.

It felt like a, well I’m not going to say it was a bed of roses, but there were no, no major problems. It was much worse when I came down to the ward and some of the anesthesia had begun to wear off completely. And it started to hurt. That’s when it became significantly worse. It hurt as soon as you moved and tried to move yourself. . . —P13

## Discussion

The aim of the present study was to explore patient experiences of mobilization out of bed to sit in a chair immediately after surgery. The findings are illustrated by the overarching theme “to do whatever it takes to get home earlier.” Mobilization was experienced as an important part of postoperative care and something the participants aimed for to enhance their recovery and discharge from the hospital. Consequently, participants never protested against early mobilization. They experienced that mobilization had a positive impact on their wellbeing, physically as well as mentally, not only in the immediate phase, but also over time. Hence, the participants requested mobilization in their continued care.

One of the most important findings is the participant experience of the physical and mental effects of mobilization immediately after abdominal surgery, since to our knowledge this was never investigated before. The participants could clearly describe easier breathing while sitting in a chair compared with lying in bed, in line with the quantitative evidence on the topic; a slumped position in bed decreases the ability to take a deep breath and has a negative impact on oxygenation.^25^^,^^26^ Interaction with caregivers was easier face-to-face while in a seated position. It was also easier to drink, eat, and keep track of time. Participants also became more aware of their situation and experienced that their minds cleared up when performing “normal” tasks compared with lying in bed.

In this study, pain was not experienced as a problem or a hinderance to mobilization, possibly because patients were given instructions about the correct mobilization technique and were assisted during the entire intervention. However, perhaps they were still under the influence of perioperative anesthetics or were screened and medicated by staff in the vicinity to reduce pain and nausea. In contrast to these results, studies evaluating patients’ experiences of fast-track care after abdominal and orthopedic surgery showed that pain, nausea, and fatigue were factors that sometimes prevented postoperative mobilization.^11^^,^^12^^,^^27^ Although, 1 study reported that pain was manageable or less than expected in the early postoperative period after colorectal surgery, but these patients found that pain still influenced their ability for physical activity.^7^

Mobilization was experienced as a way for participants in the present study to regain control of their own body after surgery, thus, increasing their autonomy in managing pain and nausea and reducing the risk for postoperative complications. These findings were similar to those in previous studies exploring patient experiences of the postoperative period after elective joint replacement surgery as well as after abdominal surgery, where patients found that getting out of bed created a sense of meaningfulness and made them more active in their own recovery.^7^^,^^8^^,^^10^^,^^11^^,^^13^ This perhaps explains why mobilization was described by the participants in the present study as an important part of their care—a goal to achieve—that would help them go home earlier. Going home was the most important goal for most of the patients in the present study.

Patients are in need of individualized information about the process they are going through to be able to take control of their situation.^9^^,^^13^^,^^14^ In addition to that, participants in the present study pointed out the importance of getting information in advance about the procedure and especially about how their body could react to mobilization. This, in combination with always having competent caregivers nearby prior to, during, and after mobilization, made the participants feel safe with the intervention regardless of physical issues such as a low blood pressure or nausea, since such issues were handled immediately. Mental preparedness, information, safety, and competent caregivers have been identified as important factors in facilitating mobilization when interviewing patients who have been through elective abdominal surgery and elective hip- and knee joint replacement surgery.^9^^,^^11^^,^^13^^,^^14^ Engagement, always being available, trust, and knowing what the patient wants without asking have emerged as important factors for patients who have commented on their experiences of nursing, quite similar to our findings.^7^^,^^9^^,^^28^

In the category “experiences and motivational factors,” the participants said that preunderstanding, attitudes, and motivational factors had an impact on their thoughts about mobilization, similar to results from a systematic review including 11 qualitative studies exploring patient experiences of enhanced recovery after surgery.^12^ Moreover, the ability to influence their physical status themselves, to increase their autonomy, and to be discharged from the hospital as quickly as possible were listed as strong motivational factors by the participants, as confirmed in previous studies.^7–9^^,^^11^

An interesting finding was that mobilization was not in the scope of focus for some participants. Their main focus was dealing with their cancer diagnosis and the results of the surgery, findings that were also confirmed in previous studies, where the underlying cause of surgery, the cancer, was associated with anxiety during the first period after surgery.^7^^,^^10^ Perhaps this can explain why patients have difficulties focusing on and following instructions during their postoperative care; their focus is elsewhere. Repeating information and instructions and the normalization of daily activities, such as getting out of bed, sitting in a chair, and having a face-to-face conversation, are important.

Some participants could not recall that they had been mobilized at the postoperative recovery unit. This is interesting in that one could argue that this was the delayed impact of anesthesia, which perhaps blurred their memories, or postoperative cognitive dysfunction.^29^

Additional information not related to the aim of the present study emerged during the analysis of the interviews. However, this information is important to present since many of the participants brought up the subject themselves. The participants experienced a gap between the postoperative recovery unit and the surgical ward. They said that they had less pain and nausea at the postoperative recovery unit and felt more attended to there than at the surgical ward.

### Strengths and Limitations

All researchers discussed, validated, and agreed on the subcategories and categories and merged them into the overarching theme. Memos were used to keep track of decisions during the entire process. All of this was done to increase credibility, dependability, and transferability.^15^^,^^16^^,^^23^^,^^24^ One strength of the study was that the research group consisted of a multidisciplinary team, all experienced in the field of research but with different perspectives and experiences in mobilization of patients immediately after elective abdominal surgery. These varied approaches and experiences enriched the analysis.

A purposeful sampling of participants was used to achieve as broad a variation of experiences as possible; a heterogenous population increases the transferability of findings.^21^ When it comes to sample size and saturation in qualitative research, there are different approaches to it in the literature. However, the most important is to assure that the data presents appropriate information, with enough richness and quality, sampled to answer the research question.^17^^,^^21^ In the present study, this was achieved by having a dialogue between the researchers and the interviewer about the quality of the interviews and the richness of the data while the study was ongoing.^21^^,^^24^ When saturation was reached, the recruitment of participants ended. However, the richness of the data can also be related to the quality of the questions in the semi-structured interview guide along with the experience of the interviewer. In present study, the interviewer was experienced in the technique. Moreover, it could be argued that the participants represented a population that was positive towards early mobilization since they were recruited from mobilization intervention in an RCT. Despite this, both positive and negative attitudes and experiences emerged during the interviews.

The mobilization interventions were part of an RCT and were performed in accordance with a pre-set protocol. Consequently, there were set routines for the mobilization at the postoperative recovery unit; for example, the patient was to be assisted by 2 caregivers during the transferring part of the mobilization. It is possible that this resulted in a feeling of safety for the patients, which could have had an impact on their experiences of early mobilization.

A strength of this study is that all interviews, except for 1, were performed within 1 to 4 days after the intervention, thus decreasing the risk of confusing memories from the 2 wards. However, additional comments on mobilization and pain in the gap/transition between the 2 wards did appear, and we found it relevant to present them. Despite that these comments are not directly connected to the aim of the study, they are valuable to explore in future studies.

The purpose of the present study was not to generalize results but to determine how patients in a particular context experience mobilization immediately after elective abdominal surgery. Whether the findings are transferable to other settings or contexts, based on similarities with other settings, samples, environments, and contexts, is up to the reader to determine.^15^^,^^19^^,^^30^ The results should be interpreted in light of this.

“To do whatever it takes to get home earlier” represents the overarching theme of participant experiences of mobilization out of bed immediately after abdominal surgery. Mobilization was experienced as an important part of postoperative care, and participants were motivated by the fact that mobilization could enhance their recovery and bring about an earlier discharge home. Participants said that mobilization had an impact on their well-being, physically as well as mentally, immediately and over time, during their continuous care at the hospital. Caregiver support and competence along with information about the process were identified as important factors in making participants feel safe and secure with the intervention.
